# Subarachnoid Hemorrhage in Mechanical Thrombectomy for Acute Ischemic Stroke: Analysis of the STRATIS Registry, Systematic Review, and Meta-Analysis

**DOI:** 10.3389/fneur.2021.663058

**Published:** 2021-05-25

**Authors:** Hubert Lee, Ayman M. Qureshi, Nils H. Mueller-Kronast, Osama O. Zaidat, Michael T. Froehler, David S. Liebeskind, Vitor M. Pereira

**Affiliations:** ^1^Division of Neuroradiology, Joint Department of Medical Imaging, Toronto Western Hospital, Toronto, ON, Canada; ^2^Department of Neurosurgery, Stanford University School of Medicine, Stanford, CA, United States; ^3^Lysholm Department of Neuroradiology, National Hospital for Neurology and Neurosurgery, UCLH NHS Foundation Trust, London, United Kingdom; ^4^Advanced Neuroscience Network/Tenet South Florida, Delray Beach, FL, United States; ^5^Neuroscience Institute, St Vincent Mercy Medical Center, Toledo, OH, United States; ^6^Department of Neurology, Vanderbilt University Medical Center, Nashville, TN, United States; ^7^Department of Neurology, University of California, Los Angeles, Los Angeles, CA, United States; ^8^Therapeutic Neuroradiology & Neurosurgery, St. Michael's Hospital, Toronto, ON, Canada

**Keywords:** subarachnoid hemorrhage, endovascular therapy, thrombectomy, stent retriever, direct aspiration, large vessel occlusion, ischemic stroke

## Abstract

**Background:** The indications for mechanical thrombectomy in acute ischemic stroke continue to broaden, leading neurointerventionalists to treat vessel occlusions at increasingly distal locations farther in time from stroke onset. Accessing these smaller vessels raises the concern of iatrogenic subarachnoid hemorrhage (SAH) owing to increasing complexity in device navigation and retrieval. This study aims to determine the prevalence of SAH following mechanical thrombectomy, associated predictors, and resulting functional outcomes using a multicenter registry and compare this with a systematic review and meta-analysis of the literature.

**Methods:** Data from STRATIS (The Systematic Evaluation of Patients Treated with Neurothrombectomy Devices for Acute Ischemic Stroke) registry were analyzed dichotomized by the presence or absence of SAH after thrombectomy. Only patients with 24-h post-procedural neuroimaging were included (*n* = 841). Multivariable logistic regression was performed to identify significant predictors of SAH. A systematic review and random-effects meta-analysis was also conducted in accordance with the PRISMA (Preferred Reporting Items for Systematic Reviews and Meta-Analysis) protocol.

**Results:** The prevalence of post-thrombectomy SAH was 5.23% in STRATIS with 15.9% (1.84% overall) experiencing neurological decline. Distal location of vessel occlusion (OR 3.41 [95% CI: 1.75–6.63], *p* < 0.001) and more than 3 device passes (OR 1.34 [95% CI: 1.09–1.64], *p* = 0.01) were associated with a higher probability of SAH in contrast to a reduction with administration of intravenous tissue plasminogen activator (tPA) (OR 0.48 [95% CI: 0.26–0.89], *p* = 0.02). There was a trend toward a higher discharge NIHSS (8.3 ± 8.7 vs. 5.3 ± 6.6, *p* = 0.07) with a significantly reduced proportion achieving functional independence at 90 days (modified Rankin Score 0–2: 32.5% vs. 57.8%, *p* = 0.002) in SAH patients. Pooled analysis of 10,126 patients from 6 randomized controlled trials and 64 observational studies demonstrated a prevalence of 5.85% [95% CI: 4.51–7.34%, *I*^2^: 85.2%]. Only location of vessel occlusion was significant for increased odds of SAH at distal sites (OR 2.89 [95% CI: 1.14, 7.35]).

**Conclusions:** Iatrogenic SAH related to mechanical thrombectomy is more common with treatment of distally-situated occlusions and multiple device passes. While low in overall prevalence, its effect is not benign with fewer patients reaching post-procedural functional independence, particularly if symptomatic.

## Introduction

Mechanical thrombectomy is well-established as the standard of care for treatment of acute ischemic stroke secondary to a large vessel occlusion (1–5). Despite demonstrating higher rates of revascularization compared to best medical management, these procedures harbor a small, but real risk, of subarachnoid hemorrhage (SAH) (6, 7). The suspected mechanisms include inadvertent microwire perforation, tearing of arterioles or venules, alterations in vascular permeability, or reperfusion injury. SAH can be seen on post-procedural imaging despite the absence of visualized vessel perforation or contrast extravasation on periprocedural digital subtraction angiography (6, 8). Based on the findings of several case series, the clinical course of isolated SAH is seemingly benign (6, 9–12). With technological advancements in endovascular devices, neurointerventionalists are pursuing more distal occlusions in medium-sized vessels achieving successful reperfusion (thrombolysis in cerebral infarction score (TICI) 2b/3) in 54–83% of cases (13–16). Accessing and retrieving devices from more distal and narrower vessels raises the concern for iatrogenic hemorrhage including SAH.

A limited number of studies have investigated clinical and procedural risk factors associated with SAH following mechanical thrombectomy (6–8, 12). They identified use of intracranial angioplasty, greater number of device passes, longer distal positioning of a stent retriever within a M2 segment branch, severe vasospasm in the involved vessel prior to thrombectomy, longer interval between stroke onset to recanalization, and longer procedural duration as significant predictors (6–8). These studies are limited by small sample sizes as well using techniques and devices that are becoming progressively dated. The objective of this study was to use the Systematic Evaluation of Patients Treated with Neurothrombectomy Devices for Acute Ischemic Stroke (STRATIS) registry, a prospective and multicenter cohort, to characterize the prevalence of thrombectomy-related SAH, its related risk factors, and impact on functional outcome. A systematic review of the literature was also performed for comparison.

## Methods

### STRATIS Registry

#### Study Design and Participants

STRATIS was a prospectively-maintained registry of mechanical thrombectomies performed with the Solitaire and Mindframe Capture Low Profile Revascularization Devices (Medtronic, Minneapolis, MN, USA) at 55 US centers from August 2014 to June 2016. The Solitaire was predominantly used as the first thrombectomy device accounting for 96.9% of procedures. The objective of this initiative was to capture real-world outcomes treating acute ischemic stroke due to large vessel occlusion. Patients were included if they presented with a National Institutes of Health Stroke Scale (NIHSS) ≥8 and ≤30, pre-stroke modified Rankin Scale (mRS) score ≤1, and mechanical thrombectomy within 8 h of stroke onset. Those participating in any multicenter randomized controlled trial (RCT) were excluded. A total of 984 patients were enrolled with the maximum number that could be contributed from a single-center limited to 75. Complete details of the study design have been previously published (17).

#### Predictors and Outcomes

Data from the registry was collected on patient comorbidities, stroke presentation, as well as procedural details. Clinical characteristics included age, sex, existing diagnoses (hypertension, diabetes mellitus, coronary artery disease, and smoking status), presenting neurological status assessed by the NIHSS, and administration of intravenous tissue plasminogen activator (tPA). Radiological characteristics of interest were presenting infarct burden on CT based on the Alberta stroke program early CT score (ASPECTS) and location of vessel occlusion. The middle cerebral artery (MCA) was further divided into segments from M1 to M3. Occlusions at the MCA M2 segment or beyond were classified as distal and those involving the internal carotid artery terminus, MCA M1 segment, vertebral artery, and basilar artery considered proximal. Procedural characteristics included type of anesthesia (conscious or general), number of device passes, use of rescue devices, and total procedural time.

The primary outcome of interest was SAH following mechanical thrombectomy. This was diagnosed by non-contrast CT as hyperdensity within the subarachnoid space or MRI brain as decreased signal intensity in the subarachnoid space on gradient echo T2^*^ sequences which were performed 24 ± 8 h post-procedure. All imaging, including mechanical thrombectomy angiography, was assessed in an independent, blinded fashion by an imaging core laboratory. The remainder of intracranial hemorrhages seen on follow up imaging were categorized according to the Hiedelberg Bleeding Classification (HBC) (18). Symptomatic hemorrhage was defined as having an associated decline in NIHSS scale by ≥4 points. Secondary outcome measures included NIHSS at discharge and functional outcome assessed by the mRS at 90 days.

### Systematic Review and Meta-Analysis

#### Search Strategy and Study Selection

A comprehensive search of the literature was conducted through the Medline, EMBASE, and Cochrane Library databases using the OVID interface including publications up to April 2020. The concepts of subarachnoid hemorrhage, endovascular thrombectomy, stent retriever, direct aspiration, and stroke were identified in our search strategy employing controlled vocabulary (National Library of Medicine's medical subject headings—MeSH) and keywords. Only publications written in the English language were considered. The references of all included studies and relevant systematic reviews and meta-analyses were manually reviewed for other eligible articles.

**Figure 1:**
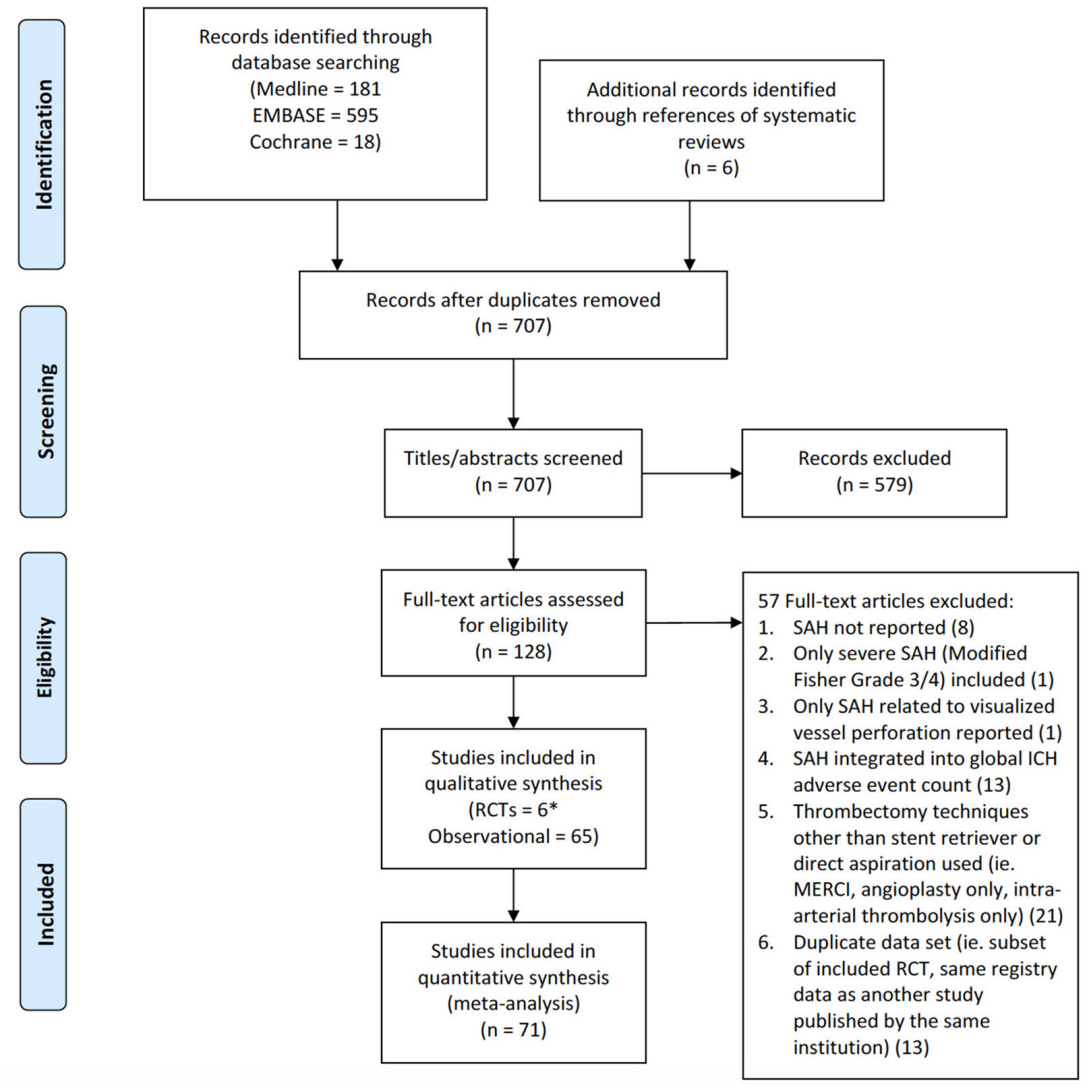
PRISMA flow diagram of study identification and selection. *For the RCT by Saver et al.—SWIFT, only one treatment arm (Solitaire stent retriever) was used.

Identified citations and their associated full text articles were reviewed by two independent investigators using pre-determined eligibility criteria after duplicates were removed using Endnote (Version X7, Thomson Reuters). The inclusion criteria applied to these studies were: (1). RCT or observational study including prospective or retrospective cohort and case control design, (2). acute ischemic stroke patients presenting with medium-to-large vessel occlusion treated with endovascular therapy, (3). adult patients (age ≥18 years). The intervention must have been performed using a stent retriever, direct aspiration catheter, or any combination of the two strategies with a post-procedural computerized tomography (CT) or magnetic resonance imaging (MRI) of the brain performed within 48 h. Systematic reviews, meta-analyses, editorials, case reports, and studies utilizing the mechanical embolus removal in cerebral ischemia (MERCI) retriever device as the initial primary treatment were excluded. The number of studies excluded from the initial screen and the number of full text articles excluded with an associated rationale are presented as a flow diagram in accordance with the preferred reporting items for systematic review and meta-analysis protocol (PRISMA) (see Figure 1).

#### Data Extraction and Risk of Bias Assessment

Data from the articles eligible for full review were extracted independently by two reviewers. The study details recorded included the first author's name, year of publication, study design, number of centers and countries involved, study duration, inclusion and exclusion criteria, and population size. Clinical, radiological, and interventional characteristics were also collected including age, sex, presenting NIHSS, presenting ASPECTS, tPA use, location of vessel occlusion, procedural anesthesia type, primary thrombectomy device used, number of device passes, procedural angiographic result (thrombolysis in cerebral infarction (TICI) score), incidence of post-procedural SAH, incidence of symptomatic SAH, and mRS at 90 days. All variables, except for study characteristics, were dichotomized based on post-procedural SAH status. The study quality of RCTs were assessed with the Cochrane risk-of-bias tool for randomized trials version 2 and the Newcastle-Ottawa Scale (NOS) or Joanna Briggs Institute (JBI) Case Series Critical Appraisal Tool used for observational studies (19, 20). Studies earning a NOS score of 6–9 were considered high quality whereas those scoring 5 or less were deemed low quality.

### Statistical Analysis

For the STRATIS database, univariate analysis between collected clinico-radiologic variables and the presence of SAH following mechanical thrombectomy was performed using the Fisher exact or chi-squared tests for categorical variables and two-sided *t*-tests for continuous variables. Predictors achieving a *p* < 0.10 underwent multivariate stepwise logistic regression with bidirectional elimination retaining only those that reached significance set at a *p* < 0.05. The contribution of each predictor to the occurrence of SAH following mechanical thrombectomy was expressed as an odds ratio (OR) with a corresponding 95% confidence interval (CI).

For the systematic review and meta-analysis, a pooled prevalence of the included studies was calculated using R statistical software [version 4.0.2, (21)] with the metafor package (22). Random effects modeling was used with the restricted maximum-likelihood estimator to account for between-study variance and individual effect sizes underwent double arcsine transformation. Sensitivity analysis was performed to identify outlying effect sizes, which was defined as an externally studentized residual >3. The associations between clinical, radiographic, and procedural variables to post-thrombectomy SAH were summarized as OR with corresponding 95% CI using the Mantel-Haenszel method and random effects model. These were performed on Review Manager software (version 5.4, The Cochrane Collaboration, Oxford, UK). Only variables with data available from 3 or more studies were analyzed. Heterogeneity was assessed for both the pooled prevalence and pooled estimates with the Higgins *I*^2^ statistic, where a value >50% was considered significant. Subgroup analysis was conducted to quantify the impact of important sources of heterogeneity with study design as the main suspected source.

## Results

### STRATIS Registry

#### Prevalence of SAH and Associated Clinical Characteristics

Post-procedural neuroimaging performed at 24 h was available in 841 of the 984 enrolled patients. A total of 44 cases of SAH were detected, 7 were symptomatic, resulting in a prevalence of 5.23% overall and 1.84% associated with neurological decline. Patients with and without SAH were similar with respect to demographics and their clinical presentation (see Table 1). Of the comorbidities investigated, diabetes mellitus was the only present in a greater percentage of patients with SAH post-mechanical thrombectomy (38.6 vs. 25.3%) however this difference only approached significance (*p* = 0.05). The location of vessel occlusion was significantly associated with occurrence of SAH with a higher frequency when the thrombus was located in the M2 segment of the MCA (36.4 vs. 15.8%) and a lower frequency at the carotid terminus (11.4 vs. 22.9%, *p* = 0.02). Several treatment factors were also related to post-mechanical thrombectomy SAH. A smaller proportion of patients with SAH received intravenous tPA (47.7 vs. 66.3%, *p* = 0.01). Clot retrieval resulting in SAH was performed with a higher percentage of device passes totaling more than 3 (20.5 vs. 9.6%, *p* = 0.02) in addition to longer mean procedural times (78.4 ± 39.3 min vs. 64.4 ± 36.9 min, *p* = 0.02).

**Table 1:** Clinical, radiographic, and procedural characteristics in STRATIS (*N* = 841).

**Mean ± SD [N], Median (IQR), or % (n/N)**	**No SAH**	**SAH**	***P*-value**
Age (years)	68.1 ± 15.0 [797] 69.6 (59.3–79.7)	70.3 ± 14.2 [44] 68.0 (61.4–81.7)	0.342
Female	46% (366/797)	55% (24/44)	0.264
**Comorbidities**			
Hypertension	73% (581/797)	70% (31/44)	0.723
Diabetes mellitus	25% (202/797)	39% (17/44)	0.050
Coronary artery disease	27% (216/797)	30% (13/44)	0.723
Smoking			0.650
Current	21% (167/797)	25% (11/44)	
Former	26% (210/797)	18% (8/44)	
Never	43% (342/797)	48% (21/44)	
Unknown	10% (78/797)	9% (4/44)	
**Preprocedural**			
Baseline NIHSS	17.3 ± 5.5 [797] 17.0 (13.0–22.0)	17.3 ± 5.8 [44] 16.0 (13.0–22.5)	0.983
ASPECTS	8.2 ± 1.6 [699] 8.0 (8.0–9.0)	8.1 ± 1.9 [40] 9.0 (7.5–9.0)	0.849
IV t-PA Use	66% (528/796)	48% (21/44)	0.012
**Location of vessel occlusion**			0.018
ICA terminus	23% (181/790)	11% (5/44)	
MCA (M1)	56% (443/790)	52% (23/44)	
MCA (M2)	16% (125/790)	36% (16/44)	
MCA (M3)	0% (2/790)	0% (0/44)	
Vertebral artery	0% (1/790)	0% (0/44)	
Basilar artery	5% (36/790)	0% (0/44)	
PCA	0% (2/790)	0% (0/44)	
**Thrombectomy**			
General anesthesia	33% (234/703)	40% (16/40)	0.382
Number of passes			0.021
≤ 3	90% (714/790)	80% (35/44)	
>3	10% (76/790)	20% (9/44)	
Rescue therapy	11% (85/790)	16% (7/44)	0.289
Procedure time (minutes)	64.4 ± 36.9 [777] 56.0 (38.0–81.0)	78.4 ± 39.3 [44] 75.0 (51.5–93.5)	0.015

*NIHSS, National Institutes of Health Stroke Scale; ASPECTS, Alberta Stroke Program Early CT Score; IV-tPA, intravenous tissue plasminogen activator; ICA, internal carotid artery; MCA, middle cerebral artery; PCA, posterior cerebral artery*.

#### Predictors of SAH

A total of 5 variables were highly associated with post-thrombectomy SAH based on a pre-determined *p* < 0.10. This included diabetes mellitus, administration of intravenous tPA, location of vessel occlusion, number of device passes, and total procedural time. The occluded vessel segment was further categorized as M2 and M3 vs. carotid terminus and M1 (excluding all posterior vessels) as well as M2 and M3 vs. all other sites except the posterior cerebral artery (PCA) to analyze the impact of distality in the anterior circulation and overall, respectively. This was necessary as the PCA data (overall few at 2/834) was not specific to the exact segment involved. The length of the procedure was analyzed in increments of 10 minutes of additional time.

Following multivariate logistic regression of these predictors, only intravenous tPA use, location of vessel occlusion, and number of device passes remained significant (see Tables 2, 3). Mechanical thrombectomy of a distally-situated thrombus had a greater odds of periprocedural SAH in the anterior circulation (OR = 3.18 [95% CI: 1.63–1.68], *p* < 0.001) and when vertebrobasilar occlusions were included (OR = 3.41 [95% CI: 1.75–6.63], *p* < 0.001).

**Table 2:** Predictors of post-thrombectomy SAH—multivariate logistic regression for anterior circulation vessel occlusions.

	**Univariate**			**Multivariate**		
	**OR**	**95% CI**	***P*-value**	**OR**	**95% CI**	***P*-value**
Diabetes mellitus	1.85	(0.99, 3.47)	0.054	–	–	–
Location of vessel occlusion: proximal vs. distal^*^	2.81	(1.48, 5.34)	0.002	3.18	(1.63, 6.18)	<0.001
IV t-PA Use	0.46	(0.25, 0.85)	0.013	0.46	(0.25, 0.85)	0.014
Number of passes	1.27	(1.04, 1.55)	0.017	1.34	(1.09, 1.64)	0.005
Procedure Time (10 min)	1.08	(1.02, 1.16)	0.016	–	–	–

*Variables no longer significant following multivariate logistic regression are denoted by “–”. IV-tPA, intravenous tissue plasminogen activator; OR, odds ratio; CI, confidence interval*.

* *Only anterior circulation vessel occlusions were included comparing internal carotid artery terminus and middle cerebral artery (MCA) M1 (proximal) to MCA M2 and M3 (distal)*.

**Table 3:** Predictors of post-thrombectomy SAH—multivariate logistic regression for anterior and posterior circulation vessel occlusions.

	**Univariate**			**Multivariate**		
	**OR**	**95% CI**	***P*-value**	**OR**	**95% CI**	***P*-value**
Diabetes mellitus	1.85	(0.99, 3.47)	0.054	–	–	–
Location of vessel occlusion: proximal vs. distal^*^	2.97	(1.56, 5.66)	<0.001	3.41	(1.75, 6.63)	<0.001
IV t-PA Use	0.46	(0.25, 0.85)	0.013	0.48	(0.26, 0.89)	0.020
Number of passes	1.27	(1.04, 1.55)	0.017	1.34	(1.09, 1.64)	0.005
Procedure Time (10 min)	1.08	(1.02, 1.16)	0.016	–	–	–

*Variables no longer significant following multivariate logistic regression are denoted by “-”. IV-tPA, intravenous tissue plasminogen activator; OR, odds ratio; CI, confidence interval*.

* *Comparison of proximal (internal carotid artery terminus, middle cerebral artery (MCA) M1, vertebral artery, basilar artery) to distal (MCA M2 and M3) vessel occlusions*.

#### Outcomes Following SAH

The mean NIHSS at discharge was not statistically different between the two groups but trended toward a higher value in patients with SAH (8.3 ± 8.7 vs. 5.3 ± 6.6, *p* = 0.07). This is despite both cohorts having nearly identical presenting NIHSS prior to mechanical thrombectomy. Having a periprocedural SAH was associated with a reduced rate of functional independence (mRS 0–2: 32.5 vs. 57.8%, *p* = 0.002) and greater fatality rate (mRS 6 30 vs. 14.5%) at 90 days (see Table 4). Of the 12 fatal cases of SAH, 4 were symptomatic post-operatively with neurological decline. No patients with symptomatic SAH or SAH associated with parenchymal hemorrhage achieved functional independence.

**Table 4:** Procedural and functional outcomes in STRATIS.

**Mean ± SD [N], Median (IQR), or % (n/N)**	**No SAH**	**SAH**	***P*-value**
Final reperfusion (TICI 2b/3)	93% (732/788)	91% (40/44)	0.620
NIHSS at discharge	5.3 ± 6.6 [620] 3.0 (1.0–7.0)	8.3 ± 8.7 [31] 4.0 (2.0–17.0)	0.070
mRS at 90 days			0.018
0	21% (157/737)	15% (6/40)	
1	23% (168/737)	8% (3/40)	
2	14% (101/737)	10% (4/40)	
3	13% (96/737)	20% (8/40)	
4	10% (76/737)	8% (3/40)	
5	4% (32/737)	10% (4/40)	
6	15% (107/737)	30% (12/40)	
mRS 0-1 at 90 days	44% (325/737)	23% (9/40)	0.007
mRS 0-2 at 90 days	58% (426/737)	33% (13/40)	0.002
**Adverse events (within 24 h)**			
Neurological deterioration^*^	8% (55/708)	18% (7/38)	0.020
Parenchymal hemorrhage (HBC 1c + 2)^**^	3% (22/797)	7% (3/44)	0.123
Remote intraparenchymal hemorrhage (HBC 3a)	0.1% (1/797)	0.0% (0/44)	0.814
Intraventricular hemorrhage (HBC 3b)	0.0% (0/797)	2.3% (1/44)	<0.001

*TICI, thrombolysis in cerebral infarction; NIHSS, National Institutes of Health Stroke Scale; mRS, modified Rankin Scale; HBC, Heidelberg Bleeding Classification*.

* *≥ 4 point worsening in NIHSS*.

** *Equivalent to European Cooperative Acute Stroke Study (ECASS) III PH1 and PH2*.

### Systematic Review and Meta-Analysis

#### Search Results and Risk of Bias of Included Studies

The initial search strategy identified 701 citations following removal of duplicates with an additional 6 studies included from the reference lists of relevant systematic reviews (see Figure 1). Of these, the full-text of 128 studies were reviewed in detail. Twenty-one studies were excluded as the frequency of SAH could not be determined as it was not reported at all in 8 or pooled with other intracranial hemorrhages in 13. Only SAH inferred by visualization of contrast extravasation on angiography from vessel perforation was reported in 1 article leading to removal. An additional study was discarded as it only included thick SAH. Thrombectomy techniques other than stent retrieval or direct aspiration was used in 21 citations resulting in exclusion. Lastly, 13 studies analyzed duplicated datasets as a subset of an included RCT or the same registry as an included observational study. This resulted in 71 studies consisting of 6 RCTs and 65 observational studies equating to a pooled population of 10,186 patients. For one RCT, only data from the stent retriever arm was used as the control group was treated with the MERCI retriever device (23).

The study quality was generally high across all design types. Only one RCT was considered high risk for compliance bias secondary to a 22% crossover rate of patients assigned to the standard medical therapy whose families could not accept the randomization result (24) (see Supplementary Table 1). Of the included cohort studies, most rated poorly on the NOS for comparability as thrombectomy success and safety were typically reported with unadjusted univariate analysis. Selection bias was also inherent to the design of before-and-after cohort studies further impacting their NOS score (see Supplementary Table 2). Similar to poor comparability as judged by the NOS many case series used descriptive statistics resulting in “not applicable” for assessment of statistical analysis with the JBI appraisal tool. A total of 4 case series were deemed lower quality due to incomplete description of participant and institutional demographics, which is relevant to the external validity of individual studies but likely imparts minimal effect on pooled statistics (see Supplementary Table 3).

#### Prevalence of SAH

**Figure 2:**
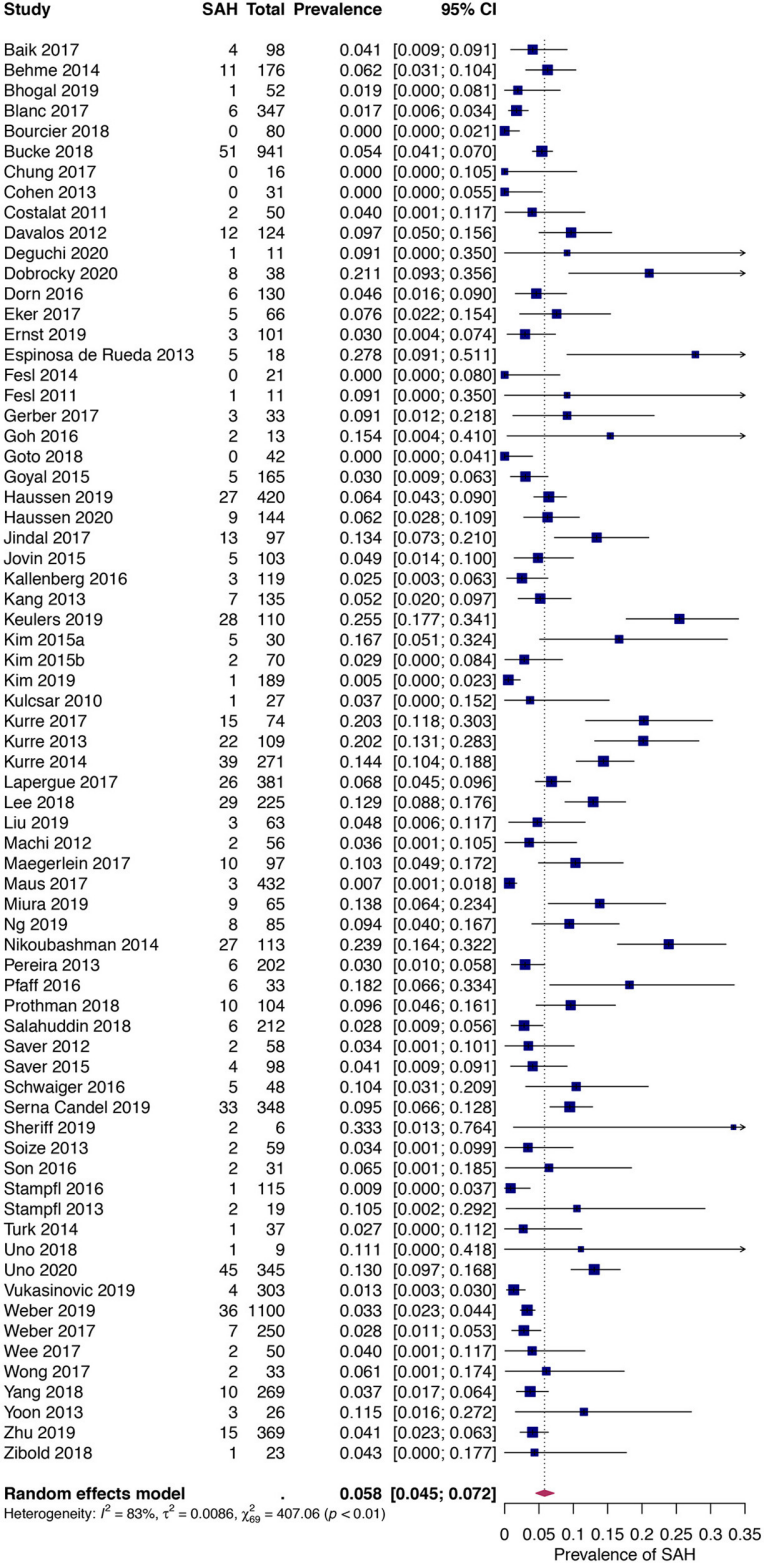
Pooled prevalence of subarachnoid hemorrhage following mechanical thrombectomy.

The pooled prevalence of SAH following thrombectomy by stent retriever, direct aspiration, or a combination of these techniques was 6.26% [95% CI: 4.75–7.93%] (655 cases in 10,186 patients). The heterogeneity was high with a Cochrane Q statistic of 407.06, *p*-value <0.0001, and *I*^2^ of 88%. Following removal of one outlying study based on pre-determined criteria (25), the prevalence reduced to 5.85% [95% CI: 4.51–7.34%] with a small improvement in the *I*^2^ to 85.2% (*p* < 0.0001) (see Figure 2). Subgroup analysis based on study design revealed a lower pooled prevalence amongst RCTs, 4.3% [95% CI: 2.75–6.11%], as compared to the cohort studies and case series, 6.55% [95% CI: 4.87–8.42%]. This difference in summary estimates approaches, but does not reach significance (*p* = 0.07). Heterogeneity was low in the RCT subgroup with an *I*^2^ of 13.57% (*p* = 0.6) but remained substantial in the observational study subgroup with an *I*^2^ of 89% (*p* < 0.0001).

#### Effect of Vessel Occlusion Location and Procedural Technique on SAH

**Figure 3:**
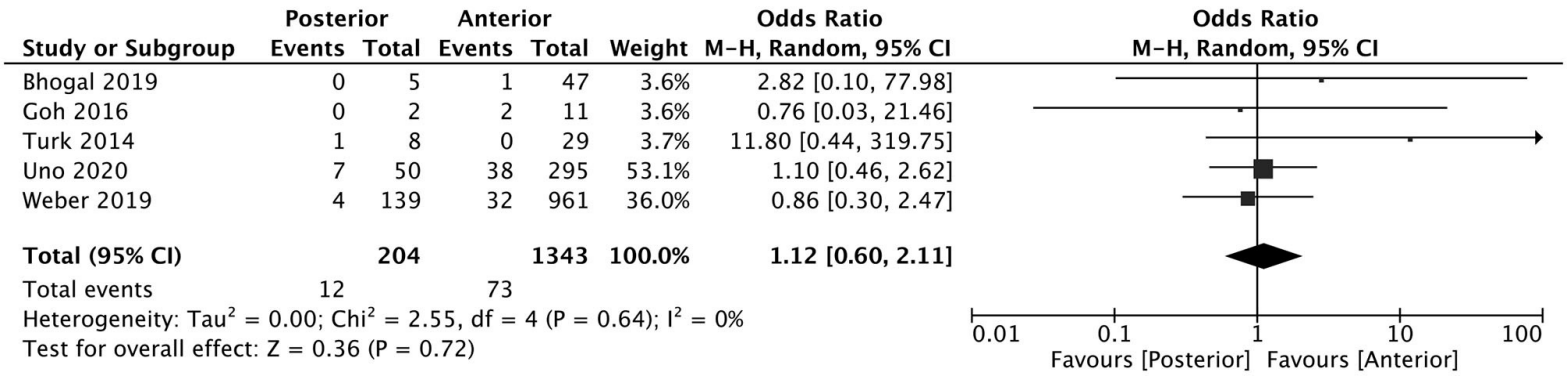
Effect of anterior vs. posterior circulation vessel occlusion on subarachnoid hemorrhage following mechanical thrombectomy.

**Figure 4:**
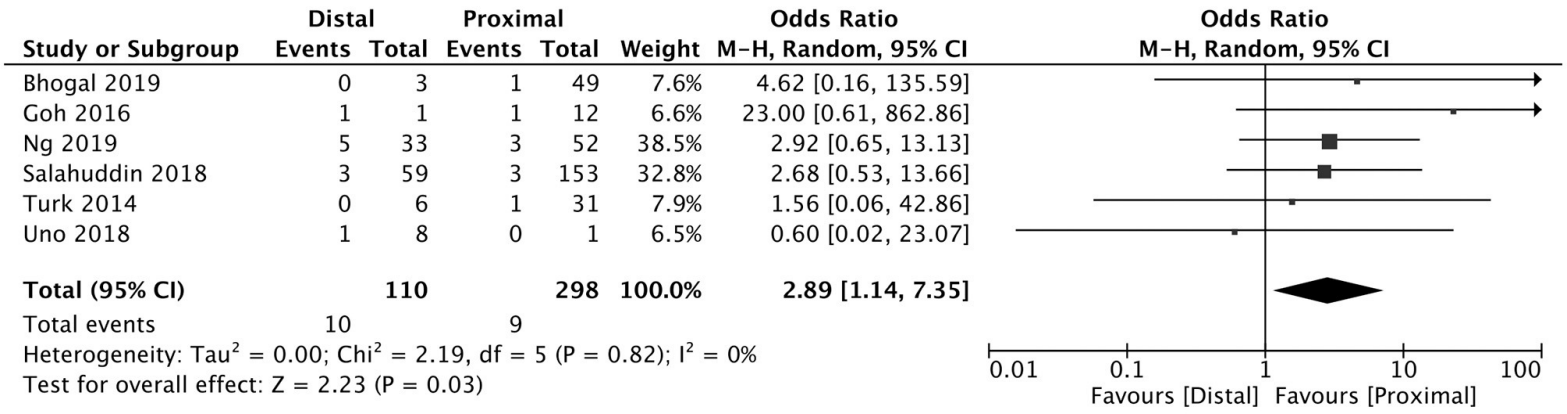
Effect of proximal vs. distal vessel occlusion on subarachnoid hemorrhage following mechanical thrombectomy.

**Figure 5:**
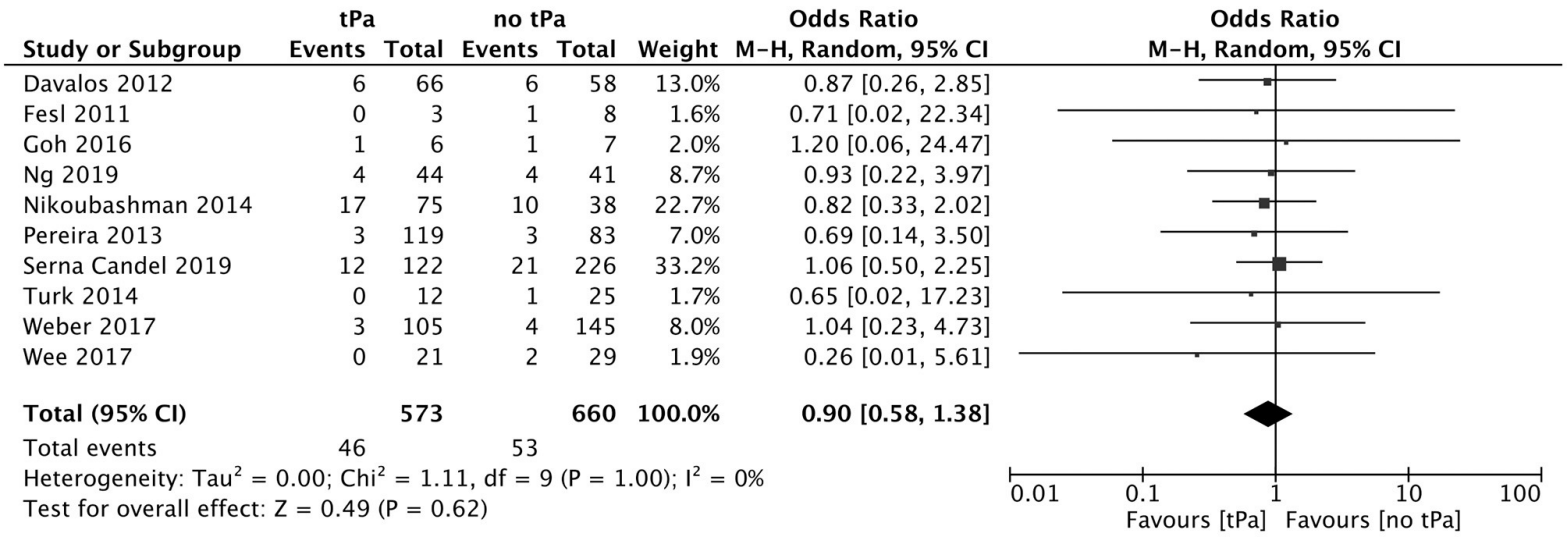
Effect of IV tPA administration on subarachnoid hemorrhage following mechanical thrombectomy.

**Figure 6:**
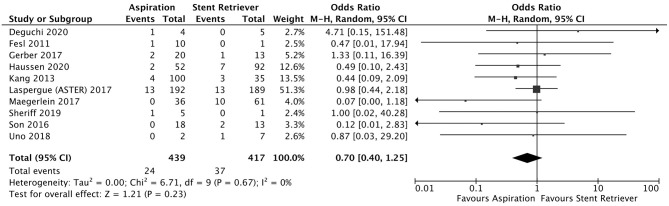
Effect of direct aspiration vs. stent retriever use on subarachnoid hemorrhage following mechanical thrombectomy.

A total of 5 studies reported data separated by internal carotid artery or vertebrobasilar involvement demonstrating similar rates of SAH following mechanical thrombectomy of the anterior circulation, 5.44%, compared to the posterior circulation, 5.88%, with an OR of 1.12 [0.60, 2.11] (*p* = 0.82, *I*^2^ = 0%) (see Figure 3). When location of vessel occlusion was characterized by distance from the Circle of Willis, distal occlusions resulted in more cases of SAH, 9.09%, than proximal occlusions, 3.02%. This difference was significant with a corresponding OR of 2.89 [1.14, 7.35] (*p* = 0.82, *I*^2^ = 0%) (see Figure 4). Pre-operative treatment with tPA in 10 studies had no significant effect on SAH occurrence compared to no tPA administration (8.03 vs. 8.03%, OR 0.90 [0.58, 1.38], *p* = 1.00, *I*^2^ = 0%) (see Figure 5). When direct aspiration was used as the first-line mechanical thrombectomy device, there was a lower rate of SAH, 5.47%, in contrast to using a stent retriever, 8.87%. However, this difference did not reach significance with an OR of 0.70 [0.40, 1.25] (*p* = 0.67, *I*^2^ = 0%) (see Figure 6). Reporting of the number of device passes was variable ranging from summary statistics (e.g., median) to categories (e.g., 3 or less vs. more than 3) preventing a meaningful quantitative analysis despite adequate number of studies.

## Discussion

SAH following mechanical thrombectomy is a known complication whose rate of occurrence has been variably reported. With the initial use of the MERCI retriever device, SAH occurred in as many as 14.1–16.4% of patients (23, 26). This was greater in frequency compared to intra-arterial thrombolysis (14.1 vs. 6.5%) (26), and later, stent retrievers (16.4 vs. 3.4%) (26). Koh et al. performed a systematic review investigating the safety and efficacy of thrombectomy using the Solitaire stent identifying 5 cases of SAH in 262 patients (1.9%) (27). A more recent pooled analysis of the Solitaire With the Intention For Thrombectomy (SWIFT) trial, SWIFT as PRIMary Endovascular Treatment (SWIFT PRIME) trial, and Study of Mechanical Thrombectomy Using Solitaire FR in Acute Ischemic Stroke (STAR) data found 2.3% of 389 patients had SAH detected on post-operative imaging (12). At the time of recruitment for these studies, mainly proximal vessel occlusions were targeted evidenced by just under 90% of the thrombectomies performed at the internal carotid terminus or first segment of the MCA. In STRATIS, 81.5% of the target anterior circulation occlusion locations were proximal resulting in a SAH prevalence of 5.23%. This is comparable to the pooled prevalence of 5.85% from the current meta-analysis. Interestingly, the combined prevalence across 6 RCTs was lower at 4.3%, particularly when compared to observational studies, perhaps due to improved operator experience or access to more modern devices. These results contrast those of Qureshi et al. who analyzed a nationwide database comparing acute ischemic stroke patients undergoing thrombectomy within or outside clinical trials finding no significant difference in the rate of SAH and intracerebral hemorrhage after adjusting for age, gender, and admission to a teaching hospital although the type of hemorrhages were combined (28). SAH resulted in neurological decline in 1.84% of STRATIS patients, which falls within the reported range (0%-7.4%) of symptomatic SAH from mechanical thrombectomy (1, 6–9).

The location of vessel occlusion, which influences the technical approach to mechanical thrombectomy, has yet to be shown as a predictor of post-procedural SAH. Weber et al. investigated 139 posterior circulation large vessel occlusions treated endovascularly (84.9% involving the basilar artery) finding this cohort consisted of more men of younger age, lower NIHSS at presentation, but comparable SAH prevalence (2.9 vs. 3.3%) when compared to anterior circulation occlusions (29). Similar results were observed in a cohort of 345 patients (50 involving the basilar artery) who underwent mechanical thrombectomy with SAH occurring in 14 vs. 12.9% in posterior and anterior circulation occlusions, respectively (30). The pooled analysis comparing anterior to posterior circulation thrombectomies identified only 3 additional smaller observational studies in the literature, not surprisingly, concluding no relationship to SAH. When the occlusion site was dichotomized as proximal or distal, there was a strong association to SAH following thrombectomy, with a higher frequency in patients with distally-located thrombus both in the STRATIS registry and our meta-analysis that included 6 observational studies (8, 31–35). Of these studies, only one independently suggested distality to be a risk factor for SAH with more instances following thrombectomy of M2-situated clots (62.5 vs. 37.7%, *p* = 0.26) and when >2 cm of the stent was deployed within a M2 branch (100 vs. 30.2% *p* = 0.002) (8). The M2 segment of the MCA is on average 1 mm (25%) smaller than the parent vessel at its origin (36, 37). These smaller diameter vessels that closely-match the outer diameter of the smallest stent retrievers and distal access catheters coupled with the sharp curves the MCA takes as it courses over the insula and operculum predispose the vessel to endothelial injury as well as neighboring arterioles and venules to higher tensile forces and possible rupture (38). Distal positioning is also more challenging to achieve having to navigate more branching points and vessel tortuosity, often without roadmap guidance, contributing to a higher risk of vessel perforation and subsequent SAH.

Greater number of passes of a thrombectomy device was found to significantly increase the prevalence of SAH in the current study. This correlates with previous studies that have included both stent retrieval and a direct aspiration first pass technique (ADAPT) in addition to other summary statistics such as the median or a differing cut-off for number of passes (7, 8, 39). The increased prevalence likely stems from compounding of the inherent risk of iatrogenic vessel injury from each revascularization attempt over repeated trials. Microperforation of the vessel wall or endothelial damage altering the blood brain barrier function may initially be minor but later exacerbated with recurrent passage of the device or subsequent reperfusion. The harm in performing multiple passes is further highlighted by a recent study showing a progressive decline in percentage of patients with good functional outcome (mRS 0–2) following mechanical thrombectomy as the number of device passes increased despite achieving a final result of good reperfusion (40).

Use of IV tPA as a bridging therapy to mechanical thrombectomy is the recommended treatment according to guidelines in the absence of contraindications however it is topic of controversy. Among the few published studies assessing its added utility, only Weber et al. reported rates of SAH finding they were similar between patients receiving IV tPA and those who did not (2.9 vs. 2.8%) (41). This absence of effect persisted when pooled with an additional 9 observational studies in our meta-analysis but contrasts the STRATIS observation of higher rates of SAH in the cohort that was not treated with IV tPA [7.9% (23/291) vs. 3.8% (21/549)] (7, 8, 32, 34, 42–46). It is difficult to rationalize this association between IV tPA administration and SAH to be causal given tPA's thrombolytic property. In fact, one of its theoretical advantages is to soften the thrombus to facilitate removal during mechanical thrombectomy promoting fewer required device passes however IV tPA use remained significant after adjustment for number of passes in the multivariate logistic regression. It could be mediated by unmeasured between-group differences in contraindications to receiving IV tPA including use of oral anticoagulants, inherent coagulopathy, or presentation outside the safe therapeutic window for intravenous thrombolysis. Of note, the mean stroke onset-to-arrival time at the recruiting center was no different when post-procedural SAH was present or absent (150.9 ± 94.9 min (*n* = 40) vs. 148.5 ± 102.6 min (*n* = 728), *p* = 0.89).

The type of anesthesia administered to patients undergoing mechanical thrombectomy has been of interest as it impacts airway protection, hemodynamic control, procedural timing, as well as patient comfort and cooperation. General anesthesia has the benefit of patient immobilization, particularly important in left-hemispheric stroke with receptive aphasia, reducing patient movements which otherwise can impair visualization and may precipitate accidental vessel injury. Randomized controlled trials have focused on determining if a difference in functional outcome exists between modalities with a meta-analysis by Schonenberger et al. including 368 patients demonstrating less disability amongst patients undergoing general anesthesia (mRS > 2: 50.8 vs. 64.9%, *p* = 0.003) with a lower frequency of intracerebral or subarachnoid hemorrhage (1 vs. 5, *not tested for significance due to low numbers*) (47). In a larger cohort of 4429 patients prospectively enrolled in the Italian Registry of Endovascular Treatment in Acute Stroke, no significant difference in SAH was seen between the general anesthesia and conscious sedation groups (3.8 vs. 2%, OR 2.230 [95% CI: 0.901–4.932]) or when compared to the local anesthesia group (3.8 vs. 2.6%, OR 1.158 [95% CI: 0.548–2.445]) after adjusting for age, sex, comorbidities, presenting NIHSS and ASPECTS, IV tPA, procedural time metrics, and thrombectomy techniques (48). This corresponds with the results of the current study and do not support a protective role of anesthesia modality against procedural SAH.

Contrary to the existing literature, SAH following thrombectomy in the STRATIS cohort had a significant deleterious effect on functional outcome. Fewer patients with SAH achieved functional independence, particularly if there was attributable neurological deterioration. This is not explained by a higher-than-normal rate of good clinical outcome in patients without SAH at 58%, which is comparable to previously published studies (2, 4, 5, 12). Raychev et al. found no significant difference in patients with and without post-thrombectomy SAH who achieved a mRS 0–2 at 90 days, however the proportion was lower in the SAH group at 44.4 vs. 55.2% (12). Similarly, a relatively reduced frequency of functional independence in patients with SAH (33.3 vs. 43.5%) was observed in the study by Yoon et al. that also did not reach significance (6). These studies had few cases of SAH, 9 and 12, respectively, which may have contributed to an underpowered analysis. The relationship between post-thrombectomy SAH, neurological decline, and resulting functional outcome is likely more complex than the presence or absence of SAH but rather dependent on factors such as thickness and distribution similar to aneurysmal SAH. The co-occurence of SAH with intraparenchymal hemorrhage has also been shown to reduce the incidence of post-thrombectomy functional independence however this data is mainly derived from series where older thrombectomy devices were used as first-line (26, 49). We found that 7% of STRATIS SAH patients also had HBC 2 (PH2) hemorrhages and all experienced poor outcomes. For comparison, Enomoto et al. reported that 18.9% (14 of 74) of patients with SAH following endovascular thrombectomy also had intraparenchymal hemorrhage but unfortunately outcome data was not included (50).

Several limitations exist in this STRATIS cohort study. Independent evaluation of radiographic features and clinical outcomes by a core lab and clinical events committee aided standardization across recruitment sites. However, variability inherent to its observational design likely remained across patient treatments including pre-interventional care, thrombectomy technique (outside the use of a Medtronic-marketed stent retriever) and post-procedural management that may be a source of confounding. Secondly, only stent retrievers were used as the primary treatment device from a single manufacturer. It is uncertain whether our results extend to stroke patients treated with ADAPT particularly with the knowledge that distal aspiration catheters produce a different pattern of endothelial cell injury *in vitro* compared to stent retrievers (38). New stents designed specifically for thrombectomy, including an updated version of the Solitaire, have also since been introduced. Lastly, only patients undergoing mechanical thrombectomy within 8 h of stroke onset were included however the therapeutic window has since been extended to 24 h (51). Future analysis of cohorts eligible for mechanical thrombectomy beyond the 8 h timeframe including direct aspiration techniques are required to further characterize their SAH risk. Our meta-analysis results should also be interpreted knowing much of the data was pooled from observational studies. Without access to individual-level data, the possible influence of selection bias or confounding arising from choice of stroke patients for mechanical thrombectomy or the procedural technique could not be accounted for. An additional limitation lies in the non-standardized definition of subarachnoid hemorrhage across the included studies with few specifying criteria, such as Hounsfield units range on CT or use of dual energy CT to improve differentiation of contrast extravasation from hemorrhage (52). As both entities appear hyperdense on CT within the subarachnoid space, misclassification could occur and partly explain the high degree of heterogeneity between studies by contributing to variable prevalence rates. Dual energy CT was also not uniformly available for the STRATIS registry which may have influenced the observed SAH rate and the resulting outcomes if those associated with contrast vs. hemorrhage truly differ.

## Conclusions

SAH following mechanical thrombectomy occurs in a small, but not insignificant, proportion of acute ischemic stroke patients. It is associated with distally-located vessel occlusions and a higher number of thrombectomy device passes required to achieve reperfusion. The influence of bridging IV tPA is uncertain with a reduced frequency of SAH when administered. Patients with post-thrombectomy SAH have poorer clinical outcomes particularly with concurrent parenchymal hemorrhage or neurological decline. Improved reporting of SAH is required in future randomized control trials.

## Data Availability Statement

The raw data supporting the conclusions of this article will be made available by the authors, without undue reservation.

## Ethics Statement

Ethical review and approval was not required for the study on human participants in accordance with the local legislation and institutional requirements. The patients/participants provided their written informed consent to participate in this study.

## Author Contributions

VP and HL contributed to the conception and design of the overall study. The STRATIS Registry was conceived, collected, and organized by NM-K, OZ, MF, and DL. The systematic review and meta-analysis was designed and conducted by VP, HL, and AQ. Statistical analysis was performed by HL. VP, HL, and AQ contributed to data analysis and interpretation with HL completing the first draft of this manuscript with further additions by AQ and VP. All authors contributed to subsequent revisions and approve of the final version.

## Conflict of Interest

The authors declare that the research was conducted in the absence of any commercial or financial relationships that could be construed as a potential conflict of interest. The reviewer KK declared a past co-authorship with one of the authors DL to the handling Editor.
