# Supplementary material for: Subarachnoid Hemorrhage in Mechanical Thrombectomy for Acute Ischemic Stroke: Analysis of the STRATIS Registry, Systematic Review, and Meta-Analysis
Source: Front Neurol. 2021 May 25;12:663058. doi: 10.3389/fneur.2021.663058 (PMC8185211; doi:10.3389/fneur.2021.663058)
Supplement: Supplementary file 1 [file Table_1.DOCX]

**SUPPLEMENTARY MATERIAL**

**Supplementary Table 1: Risk of Bias for Included Randomized Controlled Trials Assessed with the Cochrane Risk-of-Bias Tool for Randomized Trials (Version 2).**

| **Author (Year)** | **Randomization Process** | **Deviations from Intended Interventions (Effect of Assignment to Intervention)** | **Deviations from Intended Interventions (Effect of Adhering to Intervention)** | **Missing Outcome Data** | **Measurement of Outcome** | **Selection of Reported Results** | **Overall Risk-of-Bias Judgement** |
| --- | --- | --- | --- | --- | --- | --- | --- |
| Goyal (2015) | Low | Low | Low | Low | Low | Low | Low |
| Jovin (2015) | Low | Low | Low | Low | Low | Low | Low |
| Lapergue (2017) | Low | Low | Low | Low | Low | Low | Low |
| Liu (2019) | Low | Low | High* | Low | Low | Low | High |
| Saver (2012) | Low | Low | Low | Low | Low | Low | Low |
| Saver (2015) | Some Concerns** | Low | Low | Low | Low | Low | Some Concerns |

* Crossover of 14 patients (22%) in the standard medical therapy (control) group as families could not accept the randomization result

** No description of randomization method or allocation concealment in main article or protocol but no significant between-group differences

**Supplementary Table 2: Risk of Bias for Included Cohort Studies Assessed with the Newcastle-Ottawa Quality Assessment Scale.**

| **Author (Year)** | **Selection** | **Comparability** | **Outcome** | **Total** |
| --- | --- | --- | --- | --- |
| Bucke (2018) | **** |  | ** | 6 |
| Gerber (2017) | **** |  | *** | 7 |
| Haussen (2018) | **** |  | *** | 7 |
| Haussen (2019) | **** |  | *** | 7 |
| Jindal (2017) | **** |  | *** | 7 |
| Kang (2013) | *** |  | *** | 6 |
| Keulers (2019) | **** |  | *** | 7 |
| Kim (2019) | *** |  | *** | 6 |
| Lee (2018) | **** | ** | *** | 9 |
| Maegerlein (2017) | **** |  | *** | 7 |
| Ng (2019) | **** |  | *** | 7 |
| Pereira (2013) | *** |  | *** | 6 |
| Renu (2017) | *** |  | *** | 6 |
| Serna Candel (2019) | **** | * | ** | 7 |
| Son (2016) | *** |  | *** | 6 |
| Vukasinovic (2019) | **** |  | *** | 7 |
| Weber (2019) | **** | * | ** | 7 |
| Weber (2017) | **** | * | *** | 8 |
| Wee (2017) | **** |  | *** | 7 |

**Supplementary Table 3: Risk of Bias for Included Case Series Assessed with the Joanna Briggs Institute (JBI) Case Series Critical Appraisal Tool.**

| **Author (Year)** | **Yes** | **No*** | **Unclear*** | **N/A**** |
| --- | --- | --- | --- | --- |
| Baik (2017) | 9 | 5.Complete Inclusion of Participants | - | - |
| Behme (2014) | 10 | - | - | - |
| Bhogal (2019) | 9 | - | - | 10. Appropriate statistical analysis |
| Blanc (2017) | 10 | - | - | - |
| Bourcier (2018) | 10 | - | - | - |
| Chung (2017) | 9 | - | - | 10. Appropriate statistical analysis |
| Cohen (2013) | 9 | - | - | 10. Appropriate statistical analysis |
| Costalat (2011) | 9 | - | 4. Consecutive inclusion of participants | - |
| Davalos (2012) | 7 | 8. Outcomes and follow up results reported clearly | 5. Complete inclusion of participants | 10. Appropriate statistical analysis |
| Deguchi (2020) | 7 | 10. Appropriate statistical analysis | 2. Condition measured in a standard/reliable way for all participants  3. Valid methods used to identify condition | - |
| Dobrocky (2020) | 9 | - | - | 10. Appropriate statistical analysis |
| Dorn (2016) | 10 | - | - | - |
| Eker (2017) | 10 | - | - | - |
| Ernst (2019) | 9 | - | - | 10. Appropriate statistical analysis |
| Espinosa de Rueda (2013) | 9 | - | - | 10. Appropriate statistical analysis |
| Fesl (2014) | 8 | 1. Clear inclusion criteria  7. Clear reporting of clinical information of participants | - | - |
| Fesl (2011) | 9 | - | - | 10. Appropriate statistical analysis |
| Goh (2016) | 9 | - | - | 10. Appropriate statistical analysis |
| Goto (2018) | 5 | 6. Clear reporting of participant demographics  9. Clear reporting of presenting site/clinic demographic information | 4. Consecutive inclusion of participants  5. Complete inclusion of participants | 10. Appropriate statistical analysis |
| Kallenberg (2016) | 1 | 1. Clear inclusion criteria  6. Clear reporting of participant demographics  7. Clear reporting of clinical information of participants  9. Clear reporting of presenting site/clinic demographic information | 3. Valid methods used to identify condition  4. Consecutive inclusion of participants  5. Complete inclusion of participants | 10. Appropriate statistical analysis |
| Kim (2015) | 10 | - | - | - |
| Kim (2015) | 9 | - | - | 10. Appropriate statistical analysis |
| Kulcsar (2010) | 9 | - | - | 10. Appropriate statistical analysis |
| Kurre (2017) | 8 | - | 2. Condition measured in a standard/reliable way for all participants  3. Valid methods used to identify condition | - |
| Kurre (2013) | 10 | - | - | - |
| Kurre (2014) | 9 | - | - | 10. Appropriate statistical analysis |
| Malchi (2012) | 9 | - | - | 10. Appropriate statistical analysis |
| Maus (2017) | 10 | - | - | - |
| Miura (2019) | 10 | - | - | - |
| Nikobashman (2014) | 10 | - | - | - |
| Pfaff (2016) | 7 | - | 4. Consecutive inclusion of participants  5. Complete inclusion of participants | 10. Appropriate statistical analysis |
| Prothman (2018) | 10 | - | - | - |
| Salahuddin (2018) | 10 | - | - | - |
| Schwaiger (2016) | 10 | - | - | - |
| Sheriff (2019) | 8 | 8. Outcomes/follow up results reported clearly | - | 10. Appropriate statistical analysis |
| Soize (2013) | 10 | - | - | - |
| Stampfl et al. (2016) | 7 | - | 4. Consecutive inclusion of participants  5. Complete inclusion of participants | 10. Appropriate statistical analysis |
| Stampfl et al. (2013) | 9 | - | - | 10. Appropriate statistical analysis |
| Turk (2014) | 7 | 6. Clear reporting of participant demographics  9. Clear reporting of presenting site/clinic demographic information | - | 10. Appropriate statistical analysis |
| Uno (2018) | 9 | - | - | 10. Appropriate statistical analysis |
| Uno (2020) | 10 | - | - | - |
| Wong (2017) | 9 | - | - | 10. Appropriate statistical analysis |
| Yang (2018) | 8 | - | 4. Consecutive inclusion of participants  5. Complete inclusion of participants | - |
| Yoon (2013) | 10 | - | - | - |
| Zhu (2019) | 8 | - | 4. Consecutive inclusion of participants  5. Complete inclusion of participants | - |
| Zibold (2018) | 7 | 6. Clear reporting of participant demographics  9. Clear reporting of presenting site/clinic demographic information | - | 10. Appropriate statistical analysis |

* 10 question assessment with answers of yes, no, or unclear. For “no” and “unclear” answers, the question number followed by the component assessed is listed.

** N/A assigned to question 10 regarding statistical analysis if only descriptive summaries (ie. means, median, proportions) presented.
